# Targeting BAM for Novel Therapeutics against Pathogenic Gram-Negative Bacteria

**DOI:** 10.3390/antibiotics12040679

**Published:** 2023-03-30

**Authors:** Claire Overly Cottom, Robert Stephenson, Lindsey Wilson, Nicholas Noinaj

**Affiliations:** 1 Department of Biological Sciences, Purdue University, West Lafayette, IN 47907, USA; 2 Markey Center for Structural Biology, Purdue University, West Lafayette, IN 47907, USA; 3 Purdue Institute of Inflammation, Immunology and Infectious Disease, Purdue University, West Lafayette, IN 47907, USA

**Keywords:** membrane protein, beta-barrel, multidrug resistance, protein folding, Gram-negative, bacteria, vaccine, antibiotics

## Abstract

The growing emergence of multidrug resistance in bacterial pathogens is an immediate threat to human health worldwide. Unfortunately, there has not been a matching increase in the discovery of new antibiotics to combat this alarming trend. Novel contemporary approaches aimed at antibiotic discovery against Gram-negative bacterial pathogens have expanded focus to also include essential surface-exposed receptors and protein complexes, which have classically been targeted for vaccine development. One surface-exposed protein complex that has gained recent attention is the β-barrel assembly machinery (BAM), which is conserved and essential across all Gram-negative bacteria. BAM is responsible for the biogenesis of β-barrel outer membrane proteins (β-OMPs) into the outer membrane. These β-OMPs serve essential roles for the cell including nutrient uptake, signaling, and adhesion, but can also serve as virulence factors mediating pathogenesis. The mechanism for how BAM mediates β-OMP biogenesis is known to be dynamic and complex, offering multiple modes for inhibition by small molecules and targeting by larger biologics. In this review, we introduce BAM and establish why it is a promising and exciting new therapeutic target and present recent studies reporting novel compounds and vaccines targeting BAM across various bacteria. These reports have fueled ongoing and future research on BAM and have boosted interest in BAM for its therapeutic promise in combatting multidrug resistance in Gram-negative bacterial pathogens.

## 1. Introduction

Gram-negative bacteria are classically identified as diderms characterized by the presence of both an outer membrane (OM) and an inner membrane (IM). This class of bacteria include established pathogens such as *Escherichia coli*, *Acinetobacter baumannii*, and *Klebsiella pneumoniae*, all of which contribute significantly to the current antibiotic resistance crisis facing the world [1]. The pathogenesis of these bacteria requires specialized surface-exposed proteins that are embedded into the OM. These bacterial b-barrel outer membrane proteins (β-OMPs) perform essential functions for cell survival including nutrient uptake from the environment, cell signaling, cell adhesion, and enzymatic processing, but also as virulence factors important for evading host defense systems during pathogenesis [2].

**Figure 1:**
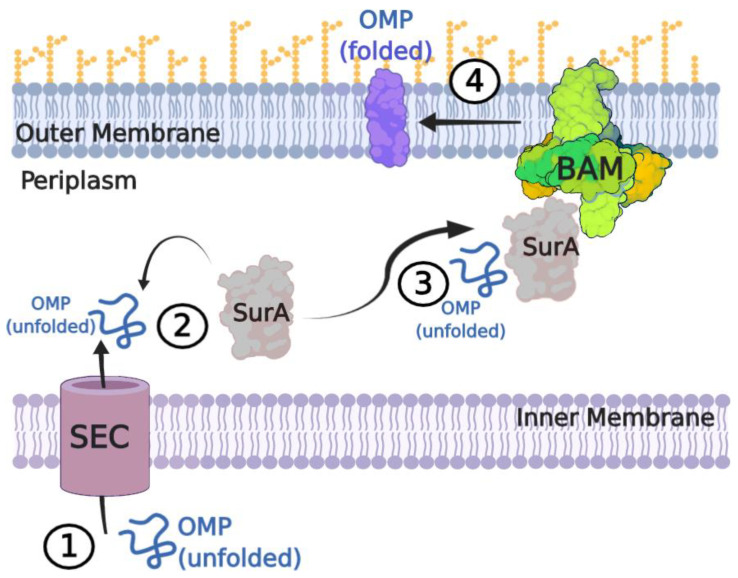
The β-OMP biogenesis pathway in Gram-negative bacteria. (1) Nascent β-OMPs are synthesized in the cytoplasm with an N-terminal signal sequence that routes them through the Sec translocon into the periplasm. (2) Periplasmic chaperones (e.g., SurA, Skp) stabilize the nascent β-OMPs to prevent misfolding or aggregation. Misfolded proteins that cannot be rescued are degraded by DegP to prevent toxicity. (3) The periplasmic chaperones then deliver the nascent β-OMPs to BAM, with SurA being the primary chaperone pathway. (4) BAM then folds and inserts the β-OMPs into the membrane using a mechanism which utilizes a hybrid-barrel intermediate with the barrel domain of BamA. This figure was prepared using BioRender.

Destined for the OM, nascent β-OMPs are first synthesized in the cytoplasm with an N-terminal signal peptide that routes them to the Sec pathway (Figure 1) [3,4,5,6]. The Sec translocon then shuttles them across the IM into the periplasm where the chaperone SurA, a peptidyl-prolyl isomerase, stabilizes and shuttles the nascent β-OMPs on to the OM for direct delivery to the β-barrel assembly machinery (BAM) for folding and insertion into the OM [7,8,9,10]. The periplasmic chaperone Skp has been shown to assist in rescuing some β-OMPs that may divert from the SurA pathway, while the periplasmic protease DegP serves to degrade misfolded β-OMPs to prevent toxicity [11,12]. A recent model postulates the formation of a super complex that spans the IM and OM to direct β-OMP assembly [13]. Here, BAM interacts with SurA, which is bound to peptidylprolyl isomerase D (PpiD), which bridges the connection to the Sec translocon. In this model, the motor protein SecA would provide the necessary energy for translocation from the IM to the OM and for membrane insertion [14].

**Figure 2:**
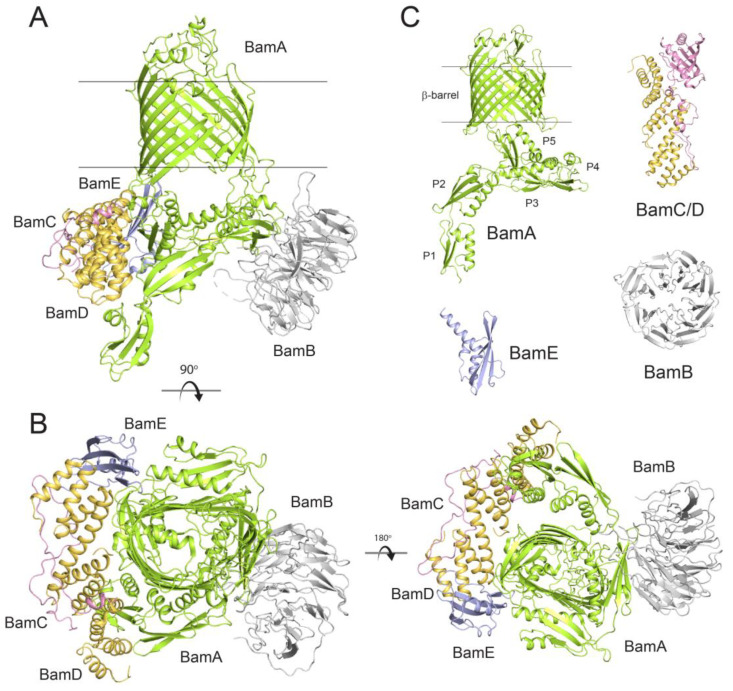
Structures of BAM and the individual Bam proteins from *E. coli*. (**A**) The structure of fully assembled BAM (PDB ID: 5D0O) shown from the side, transmembrane view; membrane boundaries are indicated by the black lines. (**B**) Orthogonal views of BAM showing the extracellular, top view (**left**) and the periplasmic, bottom view (**right**). (**C**) The individual components BamA from *N. gonorrhoeae* (PDB ID: 4K3B) showing the β-barrel domain and the POTRA domains (P1-P5), BamB from *E. coli* (PDB ID: 3Q7N), the BamC/D complex from *E. coli* (PDB ID: 3TGO), and BamE from *N. gonorrhoeae* (PDB ID: 5WAM).

The biogenesis and surface presentation of β-OMPs is achieved by BAM (Figure 2) [13]. BAM itself consists of a central β-OMP component with multiple associated lipoproteins that help fold BAM substrates into the OM. In *E. coli*, where BAM is most widely studied, the complex is composed of five components called BamA (a β-OMP), BamB, BamC, BamD, and BamE (lipoproteins) [15]. Over the past decade, multiple structures of BAM from *E. coli* have been reported in differing conformational states, providing a wealth of details about its dynamic nature and mechanism [16,17,18,19,20,21,22,23]. *E. coli* BAM has a mass of ~200 kDa with physical dimensions of ~120 Å in length, ~100 Å wide, and ~140 Å in height. Given the essential roles that β-OMPs play across all species of Gram-negative bacteria, BAM is highly conserved, although its overall composition can vary across bacterial species as will be discussed later.

BamA is the most conserved subunit of BAM and consists of an N-terminal set of polypeptide transport-associated (POTRA) domains followed by a C-terminal membrane-embedded β-barrel domain (Figure 2C) [24,25]. In *E. coli*, BamA has five POTRA repeat domains that mediate direct interaction with the lipoproteins BamB–E on the periplasmic face of the OM. BamA is the only component of BAM that is also conserved in higher eukaryotes; it is found in mitochondria (Sam50) and in plastids such as chloroplasts (Toc75 and Oep80) [26,27,28]. BamB interacts primarily at the hinge region of POTRA domains 2 and 3. BamC associates almost exclusively with BamD, which itself binds primarily along POTRA5 of BamA, but also with POTRA2. BamE is positioned between POTRA5 of BamA and BamD, with a few minimal contacts with BamC. The reported structures of BAM have supported a large dynamic complex which can be found in primarily two major conformations, termed inward-open and outward-open [29,30,31]. These conformations appear to be dictated by the conformational state of the barrel domain of BamA, where lateral opening and physical opening of the barrel seam has been shown to be required for function [32]. The POTRA domains are known to be flexible and dynamic and change conformations as BamB–E associate to form a ring-like structure on the periplasmic face where substrate handoff occurs from chaperones [17]. BAM and its components then coordinate to orchestrate the biogenesis of the β-OMPs into the OM, likely through a series of conformational changes ushering the substrate β-OMP from the periplasm to the site of insertion at the lateral seam of BamA.

BamA is the central component of BAM that is mostly widely identified as being surface-exposed and is therefore a promising therapeutic target for both small molecules and vaccines. However, several studies have demonstrated that other components of BAM can also be found on the surface, although what role they serve or how they are being translocated across the OM remains unknown. In *E. coli*, the C-terminal domain of BamC, consisting of two helix-grip domains, was convincingly reported to be surface-exposed, which has been verified in other studies [33,34]. Additionally, BamD was found to be surface exposed in wild type *N. gonorrhoeae*, with BamE being presented on the surface in modified strains lacking BamD [35]. While the mechanism for how BAM functions is still not fully resolved, these studies provide strong justification for extending therapeutic targeting to all the components of BAM and to other important proteins along the β-OMP biogenesis pathway.

Given the essential role of BAM in mediating β-OMP biogenesis in Gram-negative bacteria, and the fact that these OMPs serve essential functions for the bacteria including virulence, there is increasing excitement in targeting BAM for therapeutics to combat multidrug resistance in bacterial pathogens. Importantly, since BAM (more specifically BamA) is conserved across bacterial species, there is promise for the development of broad-spectrum novel antibiotics which can treat a wide range of bacterial infections. In this review, we present recent studies reporting novel compounds and vaccines targeting BAM across various bacteria. These reports have fueled ongoing and future research on BAM and have boosted interest in BAM for its therapeutic potential.

## 2. Mutations in BAM Demonstrate Druggability and Enhancement of Antibiotic Sensitivity

Since the discovery of BAM and its essential role in β-OMP biogenesis in Gram-negative bacteria, many studies have been conducted to determine the effect of mutations within individual Bam components on antibiotic sensitivity (Table 1). These studies provide a targeted framework to demonstrate the druggability of BAM and its individual components. An exhaustive library of mutations in BamA–E have been shown to alter bacterial resistance to various antibiotics, including vancomycin and rifampin. Understanding the underlying effect of each of these mutations on BAM function and subsequent antibiotic resistance highlights important regions in BAM that may be targeted with therapeutics and capacitates their exploitation in the battle against drug-resistant infectious agents.

BamA mutations in *E. coli* that increase the sensitivity to antibiotics include the deletion of R64, the deletion of R641-F643 or its substitution with AAA, and surface-localized insertions of HA into sites including the signal sequence, F140, Y141, Q170, N181, L231, R237, and T257 [36,37,43]. Both G807V and G807F mutations of BamA render *E. coli* more susceptible to vancomycin, and the substitution of alanine, valine, or phenylalanine at G807 increases *E. coli* sensitivity to rifampicin [44]. Similarly, in *Pseudomonas* subspecies, mutations in loop 6 of the barrel domain of BamA increase the organism’s susceptibility to bacteriocins [38]. Conversely, some BamA mutations have produced increased resistance to various antibiotics such as in *K. pneumoniae*, where the D703Y mutations in BamA increases the MIC of colistin [45]. Additionally, many BamA mutations discovered conferring resistance of *E. coli* to darobactin, including F394V, G807V, G429V, A705T, E435K, T434A, G443D, and Q445P [40]. The BamA mutation E470K increases the resistance of *E. coli* to the small molecule inhibitor MRL494 [39]. MAB1 is a bactericidal monoclonal antibody that targets BAM, and BamA mutations that confer resistance against it include E554Q, H555Y, E554Q/H555Y, V323A, P518L, T571M, G575D, and G575S [46,59].

BamA and BamD are both essential for viability in *E. coli* and other bacteria [15,64,65,66,67]. Similar to the effects of lower BamA expression, mutant *E. coli* strains expressing lower levels of BamD, *bamD_SS_* and *bamD_RBS_,* displayed increased sensitivity to bile salts [61]. Additionally, a transposon insertion at BamD codon 227 of 246 reduces protein function without eliminating it, allowing the cell to remain viable. However, the antibiotic sensitivity of this *E. coli* strain is greatly increased. Another mutant strain with decreased expression of BamD, *bamD(L13P)*, had much higher levels of unfolded β-OMPs and was more sensitive to batimastat than wild type *E. coli* [60]. Summarized in Table 1 is a list of important mutations within the essential BamA and BamD components, along with a series of non-essential mutations which alter the resistance of Gram-negative organisms to antibiotics.

## 3. Targeting BAM with Small Molecules

Several small molecules targeting BAM have been reported recently (Table 2). In one study, the small molecule VUF15259 was discovered while developing a reporter assay to screen for defects in the Gram-negative bacterial autotransporter (AT) pathway [51]. VUF15259 induced cell envelope stress and inhibited the processing and secretion of several ATs. Treatment of *E. coli* cells with VUF15259 produced growth defects, most notably in strains carrying gene mutations of OMP folding pathway proteins such as DegP, SurA, and BamB. VUF15259 treatment also decreased OMP levels including OmpA, OmpF, OmpC, BamA, and ectopically expressed PhoE. Additionally, VUF15259 treatment caused lower levels of BAM, contributing to altered membrane permeability [51]. Together, these data suggest that VUF15259 affects BAM function, however, it is not strongly bactericidal in *E. coli*. VUF15259 treatment did not affect the folding and insertion of OmpA into liposomes containing reconstituted BAM. Thus, further studies are needed to elucidate the exact target and mechanism of VUF15259.

Another small molecule, nitazoxanide (NTZ), has been shown to impede the Gram-negative chaperone–usher pathway in a BAM-dependent manner [68]. Previously, nitazoxanide was shown to inhibit the formation of pili, preventing uropathogenic *E. coli* from adhering to host tissues during infection [78]. Pilus formation is controlled by the chaperone–usher (CU) pathway which consists of a periplasmic chaperone protein and an OM usher protein, whose biogenesis depends on BAM [79]. By observing the effect on levels of the P Pilus usher protein PapC, a recent study showed that modulation of the BAM components conferred sensitivity or resistance to nitazoxanide in *E. coli* [68]. Overexpression of BAM increased levels of PapC in the OM and conferred resistance to nitazoxanide treatment. Additionally, reduction in BamA levels using the *bamA101* mutant strain led to decreased levels of PapC in the OM and enhanced sensitivity to nitazoxanide. Mutations in BamB and BamE, however, reduced sensitivity to nitazoxanide treatment; mutations in BamB, particularly, led to a decrease in properly folded PapC usher in the OM and an impaired ability of *E. coli* to agglutinate human blood cells, which requires properly assembled pili on the bacterial surface. Notably, nitazoxanide treatment specifically affected levels of the usher PapC in the OM but not OmpF, OmpC, or OmpA, leading to the hypothesis that BAM may play a role in the CU pathway distinctly from the mechanism it uses to fold other β-OMPs. Screening to identify mutants conferring resistance to nitazoxanide led to the discover of the P100S mutation in BamD. While the exact binding site and mechanism of action for nitazoxanide with BAM remains unknown, BamA, BamB, BamD, and BamE are all critical for nitazoxanide sensitivity of BAM. Since BAM and chaperone–usher-mediated pilus assemblies are conserved in a variety of Gram-negative pathogens, nitazoxanide treatment could be used to combat a diverse range of bacterial infections.

The discovery of MRL-494 came from the screening of a chemical library to find novel small molecules capable of inhibiting the growth of wild-type *E. coli* [39]. MRL-494, which targets BAM, inhibited the growth of wild-type *E. coli* possessing an intact OM barrier and active chemical efflux pumps. Treatment with MRL-494 decreased the levels of BamA, LptD, OmpA, OmpC, and LamB (all β-OMPs), but not of the cytoplasmic protein GroEL or the periplasmic protein DegP. Additionally, MRL-494 treatment induced the σ^E^ stress pathway, indicating the presence of unfolded β-OMPs in the periplasm. Supporting the hypothesis that MRL-494 targets β-OMP assembly, the reduction in BamA levels or the deletion of other genes involved in β-OMP biosynthesis (BamB, DegP, and SurA) led to increased sensitivity to MRL-494. Additionally, MRL-494 treatment led to decreased assembly of folded LamB at the OM with increased levels of unfolded LamB in the periplasm. Mutagenesis screening identified an MLR-494-resistant BamA mutant, BamA E470K, which is located within b4 of the BamA barrel domain. Expression of the BamA E470K mutant decreased *E. coli* sensitivity to MRL-494 in wild-type and ΔbamB backgrounds and partially rescued the proper folding of LamB. Experiments manipulating the cellular BamA allele (BamA WT/E470K) and copy number (1 vs. 2) present during MRL-494 treatment suggested that BamA was the direct target of MRL-494 action. Thermal stabilization experiments suggested that MRL-494 was binding directly to or near BamA in BAM, while the E470K mutation did not affect this binding. The protective effect conferred by the E470K mutation in BamA depended on the type of amino acid present in the mutated protein with charged residues (K/R) conferring the most resistance to MRL-494 and uncharged residues (A/G) conferring the least. A heat-modifiability assay showed that the E470K mutation reduces the stability of the BamA barrel, which was most pronounced when the altered amino acid was charged. Additionally, the E470K mutation suppressed the lethal BamA E373K mutation which impairs the ability of BamA to associate with BamD. Thus, the BamA E470K mutation may circumvent the primary BAM-mediated β-OMP assembly pathway to allow β-OMP assembly in the presence of MRL-494. As before, mutating E470 of BamA to a charged residue suppressed the lethality of the E373K mutation, while mutating to a nonpolar amino acid did not. Therefore, it was concluded that BamA E470K confers resistance to MRL-494 treatment by modulating BamA conformations and activity, and not by preventing binding of the molecule. Interestingly, MRL-494 can also kill Gram-positive bacteria through a distinct mechanism involving disruption of the Gram-positive cytoplasmic membrane.

The small molecule inhibitor IMB-H4 was discovered during compound screening focused on disrupting the BamA–BamD interaction in a yeast two-hybrid system [69]. A glutathione pull-down assay was used to demonstrate that the disruption is caused by IMB-H4 binding to BamA. Transmission electron microscopy experiments revealed a change in membrane morphology following IMB-H4 treatment and an ethidium bromide assay resulted in a corresponding increase in membrane permeability. Minimal inhibitory concentrations (MICs) of IMB-H4 spanned 4 to 32 µg/mL against clinically isolated *E. coli* strains. IMB-H4 was found to be non-toxic to eukaryotic cells and to synergize with other antibiotics including polymycin B, vancomycin, and gentamicin when tested against *E. coli*. Thus, IMB-H4 represents a promising lead compound towards targeting BAM in *E. coli* and possibly other bacterial species.

## 4. Targeting BAM with Peptides and Proteins

In addition to small molecules, several reports have studied peptides and proteins that target BAM for their potential as promising antibiotics (Table 2). One study examined the use of monoclonal antibodies (mAb) as antibiotics, focusing on the properties of a mAb called MAB1 [46]. Typically, lipopolysaccharide (LPS) serves as a barrier preventing the binding of antibodies to surface proteins in Gram-negative bacteria, however, MAB1 was identified while screening a library of mAbs against *E. coli* Δ*waaD*, a strain of *E. coli* with truncated LPS. It was discovered that MAB1 binds extracellular loop L4 of BamA in *E. coli* and is bactericidal, inducing the σ^E^ stress response and downregulating the levels of β-OMPs, but not of periplasmic proteins. Additionally, the authors discovered that the fluidity of the OM affected the efficacy of MAB1, with more rigid membranes attenuating the antibiotic activity of MAB1. While not effective against wild-type *E. coli*, MAB1 represents a proof-of-principle pipeline towards engineering antibodies, which often have excellent pharmacological properties, as putative antibiotics.

An antigen-binding fragment (Fab) of MAB1 called Fab1 is also lethal in vivo [71]. Interestingly, the Fab1–BAM complex can still accomplish substrate folding when reconstituted into liposomes, though with reduced efficiency compared to unbound BAM. The EM structure of the Fab1–BAM complex shows extracellular binding of the Fab to BamA eL4 trapping BAM in an outward-open conformation (PDB 7NCS).

Another study reported the production and characterization of nanobodies against BamA following immunization of alpacas with the barrel domain of BamA. Binding of the nanobodies to BamA was characterized by surface plasmon resonance (SPR) and nuclear magnetic resonance (NMR) spectroscopy which facilitated the selection of nanobodies for crystallization [72]. The nanobodies nanoE6 and nanoE7 were found to bind BamA in the outward-open and inward-open conformations, respectively, with minimal conformational changes observed outside of the lateral gate region of BamA. NanoE6 binds to extracellular loop L4 of BamA, while NanoE7 binds along extracellular loops L3 and L6. High-resolution NMR data were collected on the BamA–nanobody complexes which showed that, while BamA alone was dynamic in solution, each nanobody could effectively lock BamA into each respective conformation observed in the crystal structures. While the nanobodies were not tested as putative antibiotics against *E. coli* in this study, their ability to effectively lock BamA in a single conformation would indeed abrogate BAM function which would immediately lead to damaging defects in the OM and eventual cell death.

A small library of peptidomimetic antibiotics was discovered by taking advantage of elements of an β-OMP-targeting antibiotic called Murepavadin [45,80]. Synthetic cyclic peptides were screened against the ESKAPE pathogens (*Enterococcus faecium*, *Staphylococcus aureus*, *Klebsiella pneumoniae*, *Acinetobacter baumannii*, *Pseudomonas aeruginosa*, and *Enterobacter* species) and three were found to have a bactericidal effect of high potency combined with low mammalian cell toxicity. These peptide-like macrocycles are characterized by the ability to bind both LPS and BamA and to permeabilize bacterial membranes. In vitro studies confirmed the binding of the peptides to BamA via extracellular loops, and mouse model studies verified in vivo efficacy of the peptides. This promising approach has yielded several antibiotic candidates effective against current drug-resistant bacterial pathogens.

Another peptidomimetic antibiotic is JB-95, a β-hairpin macrocyclic peptide [73]. JB-95 disrupts the OM, but not the IM of Gram-negative bacteria, as demonstrated by fluorescence microscopy and transmission electron microscopy. Additionally, an assay measuring the release of periplasmic β-lactamase and cytoplasmic β-galactosidase showed the release of β-lactamase but not β-galactosidase after the treatment of *E. coli* with JB-95. JB-95 also significantly reduced the percentage of β-OMPs expressed compared to untreated *E. coli*. The reduction in β-OMPs led to the hypothesis that the mechanism of JB-95 involves the inhibition BAM activity by direct binding to BamA; however, further studies are needed to confirm this.

BamD has been shown to associate with unfolded β-OMPs in vitro such as unfolded BamA [81]. Since BamD is an essential component of BAM, a separate study probed whether BamD may serve the primary function of binding nascent β-OMPs during their biogenesis into the OM by BAM. BamA truncations were used to map the region of BamA that directly interacted with BamD [75]. Using pull-down assays, BamD was found to bind to the C-terminal region of the barrel domain of BamA consisting of 96 amino acids spanning residues 715–810. A peptide consisting of residues 715–810 of BamA, and a shortened optimized 15-residue peptide termed Peptide 2, were found to inhibit the folding of BamA. Peptide 2 was shown to competitively inhibit BAM-mediated folding of OmpA and BamA into proteoliposomes. Peptide 2 contains the conserved β-signal of BamA, which is thought to be responsible for the recognition of nascent β-OMPs by BAM. Deletion of the β-signal from the 96-residue BamA peptide abolished binding to BamD in vitro, while mutagenesis studies revealed that W776 was critical for binding of the peptide to BamD. In studies in *E. coli*, the expression of the peptide containing residues 715–810 of BamA had a toxic phenotype, which also increased the sensitivity of the cells to vancomycin and rifampin. Deletion of residues 769–776 in BamD abrogated the toxicity of the BamA 715–810 peptide when expressed in *E. coli* and partially reversed the sensitivity of cells to vancomycin and rifampin. Finally, UV-crosslinking of modified BamA peptides showed that BamD can form a cross-linked adduct with BamA at position W776, further supporting that the tryptophan within the BamA β-signal is crucial for the recognition of nascent β-OMPs by BamD. These studies postulated that BamD may serve an essential role in the recognition of unfolded β-OMPs, through their C-terminal ꞵ-signal, during delivery and folding into the OM by BAM. This poses an attractive target for the development of novel antimicrobial therapeutics given that no BamD orthologs are present in the higher eukaryotic conserved systems such as the SAM complex in mitochondria [82,83,84,85] or the TOC complex [86,87,88] and Oep80 protein in chloroplasts [26,27,28]. Therefore, compounds designed to disrupt β-OMP recognition by BamD in bacteria are less likely to have off-target effects within these orthologous systems which may be present when focusing on the conserved BamA component of BAM.

Another BAM-targeted peptide that has promise as a putative therapeutic comes from the marine sponge *Axinella donnani* [76]. Using a comparative genomics approach verified with substrate and docking analysis, it was elucidated that the receptor for this peptide was Omp85 (BamA). Using computational methods including protein–peptide docking, Rosetta modeling of the protein–peptide complex, and molecular dynamics simulations, the binding site of the 39-residue a-helical peptide was mapped back to the conserved motifs RGF and YGDG in BamA [89,90]. While a potentially exciting study, these in silico results require experimental validation to lend additional legitimacy to these findings and to determine what effect this peptide has on BAM function.

An antibiotic discovery study was fueled by the hypothesis that undiscovered antimicrobials targeting Gram-negative bacteria might be produced by bacterial symbionts found in the nematode gut microbiome. To this end, concentrated supernatants from one nematode symbiont, *Photorhabdus*, were found to indeed inhibit the growth of *E. coli*. From these supernatants, the authors identified a 966 Da hexapeptide, termed darobactin, as the inhibitory compound [40]. The darobactin structure has two cyclic linkages between the amino acids in the hexapeptide and an MIC of 2 µg/mL against drug resistant *E. coli* and *K. pneumoniae*. Mouse model experiments subsequently confirmed in vivo efficacy of the compound. The identification of a biosynthetic gene cluster (BGC) responsible for darobactin production revealed darobactin homologs in several bacteria species, including *Photorhabdus* and *Yersinia*. Passaging *E. coli* in the presence of darobactin produced several darobactin-resistant strains of *E. coli*, which were then mapped back to the lateral seam within the barrel domain of BamA. An in vitro folding assay demonstrated dose-dependent inhibition of BAM-mediated β-OMP folding by darobactin with micromolar IC_50_ values, while binding studies using isothermal titration calorimetry (ITC) showed direct binding of darobactin to BAM. NMR experiments showed that the addition of darobactin locked the barrel domain of BamA in the inward-open conformation. It was concluded that darobactin acts by locking BamA in a single conformation, thereby inhibiting its function. Subsequent structural studies reported the structure of darobactin bound to BAM, revealing that darobactin binds at the lateral seam of the barrel domain of BamA, mimicking substrate binding and preventing nascent β-OMPs from associating with BAM [41]. In other studies, the darobactin-biosynthetic gene cluster was introduced into *E. coli* in order to heterologously produce an expanded library of darobactin analogues [91]. These improved compounds were found to bind BAM similarly to the original compound; however, they boasted improved MICs over the original darobactin compound with more favorable pharmacological properties.

Searching for genes related to darobactin led to the discovery of another structurally unrelated antibiotic peptide, dynobactin A [48]. Here, a computational approach uncovered enzymes similar to darobactin A and probed their gene neighborhoods for darobactin-like propeptides. A phylogenetic tree of results was created and used to select strains to screen for antibiotic activity. The top hit compound, a water soluble decapeptide termed dynobactin A, was identified from these strains using mass spectrometry. It did not induce toxicity in human cells or mouse models at 1 mg/mL and was not active against selected Gram-positive strains. *E. coli* mutations resistant to dynobactin A were mapped to BamA and the structure of dynobactin A bound to BamA was determined by cryo-EM and by X-ray crystallography. The efficacy of dynobactin A as an inhibitor of BAM activity is more potent than darobactin, as demonstrated by nanomolar IC_50_ values. The discovery of dynobactins represents a new class of promising antibiotic natural products and an effective computational pipeline for the detection of additional antimicrobial peptides.

## 5. Bacterial Warfare Using BAM: Lectin-like Bacteriocins and Contact-Dependent Growth Inhibition

Bacteriocins are a class of proteins or peptides secreted by bacteria that have a bactericidal effect on competing bacteria. Lectin-like bacteriocins (LlpA) are a class of proteins that contain two B lectins and a C-terminal extension that binds to D-rhamnose moieties within LPS [38]. One investigation isolated *Pseudomonas fluorescens* mutants resistant to bacteriocin LlpA1 from *Pseudomonas protegens* [77]. Genome sequencing revealed that the mutations responsible for resistance were in genes responsible for LPS biosynthesis and in BamA (Table 2). Mutations conferring resistance to LlpA were mapped to the surface-exposed loops 4 and 6 within the barrel domain of BamA. Examination of multiple bacteriocins targeting different *Pseudomonas* species confirmed that single nucleotide polymorphisms present in BamA’s surface-exposed loops mediate resistance to bacteriocins. Additionally, *Pseudomonas* strains could be made resistant or sensitive to specific bacteriocins by changing which BamA allele they expressed. The loop 6 sequence determined sensitivity to specific bacteriocins, and species avoided self-killing by expressing a BamA variant with a loop 6 sequence unrecognized by their respective bacteriocin. This bacterial killing mechanism by bacteriocins targets BAM at the cell surface without requiring uptake of the bacteriocin. LlpA may act by inhibiting conformational changes in BamA, which interferes with the binding of nascent β-OMPs to BAM and prevents their biogenesis into the OM [38]. Alternatively, like darobactin, the C-terminal extension of LlpA may mimic a β-strand which binds directly to the lateral gate of the barrel domain of BamA to prevent the interaction with nascent β-OMPs. Solving the LlpA-bound BamA structure will provide mechanistic insight into LlpA’s mode of inhibition of BAM and provide valuable insight towards further development of bacteriocins as possible therapeutics against pathogenic bacteria. A second class of lectin-like bacteriocins containing one β-lectin domain, the LlpBs, has been identified in *Pseudomonas* [77]. However, although LlpB appears to also act by binding to LPS such as LlpA, more studies are needed to determine if its bactericidal activity is similarly dependent on BamA or other components of BAM.

BamA is associated with another form of bacterial warfare called contact-dependent growth inhibition (CDI) [92]. In CDI, binding between the CdiA/CdiB two partner system on an inhibitor cell and a receptor on a target cell produces growth arrest on the target cell, giving the inhibiting cell a competitive growth advantage. A genetic screen designed to find mutations that suppress CDI growth inhibition in *E. coli* found that mutations reducing the BamA expression resulted in resistance to CDI [93]. Additionally, treatment of *E. coli* with an anti-BamA antibody which binds to the extracellular domains of BamA also led to CDI resistance, presumably by disrupting binding between BamA on the target cells and CdiA on the CDI+ *E. coli*. Thus, BamA was identified as the receptor for the CdiA/CdiB two partner system in *E. coli*. Studies examining how CDI proteins from *E. coli* strain EC93 selectively inhibit the growth of similar strains of *E. coli*, but not other *Enterobacteriaceae* species, provide additional insight into the role BamA may play in CDI [94]. Although BamA is highly conserved among *Enterobacteriaceae*, sequence differences in extracellular loops 6 and 7, which comprise the CdiA binding surface and vary among *Enterobacteriaceae* species, were discovered to be responsible for *E. coli* EC93 CDI selectivity. The expression of *E. coli* BamA in other *Enterobacteriaceae* sensitizes the bacteria to *E. coli* EC93-mediated CDI, while substitution of another *Enterobacteriaceae* species’ BamA in *E. coli* confers resistance. Sequence differences in the CdiA-binding surface of BamA allow *E. coli* to discriminate between “self” and “non-self” species and to produce species-specific CDI.

Both lectin-like bacteriocins and CDI systems target BamA and demonstrate naturally occurring bacterial systems targeting essential, surface-exposed proteins that could be tapped for antimicrobial therapeutics. Sequence variations in BamA loops which differentiate between self- and non-self-bacterial species lead to selective killing in the case of *Pseudomonas* bacteriocins targeting BamA [38,95,96] and selective growth inhibition in the case of *E. coli* CDI [94]. These studies lay the foundation for the possibility that these and other systems of natural interbacterial competition may be re-engineered or hijacked in order to target bacteria in a species-specific manner.

## 6. Targeting BAM for Vaccines

The emergence of multidrug-resistant (MDR) strains of a variety of pathogenic bacteria, and the likelihood that many of these bacterial infections may soon be untreatable with existing antibiotics, drives the need for the discovery and development of other methods to combat MDR bacteria [1]. Better than antibiotics, vaccines have the potential to promote a strong host immune response and lasting protection against bacterial infections. Given that it is essential and conserved across all Gram-negative bacteria, BAM is an attractive and exciting candidate not only for antibiotic discovery, but also for the development of new vaccines.

*A. baumannii* is a clinically significant Gram-negative pathogen that causes a variety of nosocomial infections [97]. New MDR strains of *A. baumannii* are continually emerging and the World Health Organization (WHO) has named the development of novel therapeutics to combat *A. baumannii* a “critical priority” [98]. A recent study used a proteomics and bioinformatics approach to identify proteins in the *A. baumannii* proteome suitable for vaccine development [99]. Screening the *A. baumannii* proteome for candidate proteins that were essential, surface-exposed, and non-homologous to human proteins or common human gut microbiome bacterial proteins identified three: BamA, FimD, and Rhs. In silico methods predicted B-cell-derived T-cell epitopes within the three proteins, which were projected to elicit B-cell and T-cell mediated immune responses while non-allergenic to the host. A chimeric construct was made containing the three peptide epitopes from BamA, FimD, and Rhs, with the cholera toxin B subunit as an adjuvant. This chimeric peptide vaccine could bind the host innate immunity receptor TLR4 in silico as shown by molecular docking and MD simulations. This vaccine approach has potential for Tigecycline-resistant *A. baumannii* infection prevention. Moreover, its design may circumvent problems associated with other types of vaccines, such as attenuated pathogens or subunit-based vaccines, maximizing both safety to the vaccine recipient and the immune response produced. Subsequent in vitro and in vivo studies are needed to establish the clinical significance of this vaccine.

Another MDR *A. baumannii* vaccine study investigated the use of BamA in subunit vaccines, testing whether BamA could elicit a protective immune response in mice [24]. Since *A. baumannii* BamA was predicted to contain B-cell and T-cell epitopes, in silico analysis indicated that it could serve as an effective vaccine component. Purified recombinant BamA from *A. baumannii* was injected into mice with an adjuvant control; immunizations with recombinant BamA led to high titers of IgG antibodies in the host. Compared to control mice, mice injected with recombinant BamA had a significantly lower bacterial load in their lungs, lower pro-inflammatory cytokine levels, higher anti-inflammatory cytokine levels, and greater survival when challenged with a lethal dose of MDR *A. baumannii*. Both active and passive immunization with BamA led to increased survival (80% and 60%, respectively) of mice challenged with MDR *A. baumannii*. Additionally, serum from mice immunized with BamA was capable of opsonophagocytic killing of MDR *A. baumannii* in vitro. Since immunization with BamA produced a strong protective response against MDR *A. baumannii* infection in a mouse model, BamA has been established as a strong candidate for vaccine development in humans.

*Neisseria gonorrhoeae*, another drug-resistant Gram-negative pathogen, is characterized as an “urgent threat” by the WHO [100]. Multiple recent studies recognize BamA as a promising candidate for use in vaccines against *N. gonorrhoeae*. One bioinformatics study used the database PubMLST to identify different BamA alleles present in 3946 isolates of *N. gonorrhoeae* [101]. The position of SNPs or amino acid substitutions in each BamA allele was located and their prevalence calculated in different *N. gonorrhoeae* isolates. Mapping the locations of the SNPs and amino acid substitutions onto the 3D structure of *N. gonorrhoeae* BamA enabled the identification of 69 BamA different alleles across the *N. gonorrhoeae* isolates in PubMLST with 59 amino acid substitutions occurring in those BamA alleles. Several amino acid substitutions occurring in BamA alleles are present in surface-exposed regions of the protein, with two of them occurring with medium prevalence (20–30%) in the isolates surveyed. Inclusion of BamA variants containing different amino acid polymorphisms, especially at surface-exposed regions of the protein, in a subunit-based vaccine may broaden the effectiveness of the vaccine to a wider range of *N. gonorrhoeae* strains.

A proteomics-based approach similarly identified BamA as a candidate for use in vaccines against *N. gonorrhoeae* [102]. Samples for proteomics came from *Gonococcus* cultured in standard conditions or conditions mimicking human host infection. Anti-sera raised against recombinant BamA in rabbits had a bactericidal effect on the *Gonococcus*. Bactericidal BamA antibodies cross-reacted with many strains of *N. gonorrhoeae*. This work validated both a proteomics-based approach for discovering vaccine candidates against *N. gonorrhoeae* and the use of BamA as a promising target for ongoing and future vaccine development studies.

BamA has also been identified as a vaccine candidate for a wide range of other Gram-negative pathogens. In *E. coli,* BamA is a potential candidate for use in subunit vaccines against colibacillosis [103]. BamA is highly conserved in *E. coli* species and among *Salmonella* and *Shigella*, but not *Pseudomonas*, and in silico analysis predicted that BamA would be non-allergenic in humans and mice. Residues 448–810 of the barrel domain of BamA (rBamA) were expressed and purified from *E. coli* and used for mice immunizations. High titers of serum antibodies were produced against rBamA and serum from BamA-immunized mice increased the killing of *E. coli* by neutrophils in an in vitro opsonophagocytosis assay. Immunization with BamA produced a survival rate of 80% survival when mice were subsequently challenged with a lethal dose of *E. coli* versus 20% for injection vehicles only. This study demonstrates that BamA represents a strong candidate for subunit vaccines against pathogenic *E. coli*.

Additionally, a reverse- and structural-vaccinology approach identified BamA as a vaccine candidate using in silico methods in multiple studies. A bioinformatics study identified *Leptospira interrogans* proteins, which are promising vaccine candidates [104]. Lipoproteins or transmembrane β-barrel proteins conserved across pathogenic *Leptospira* species and potentially immunogenic (i.e., had MHC-II recognition epitopes) were identified. After building structural models of candidate proteins, the locations of conserved MHC-II epitopes were mapped onto the structures. The study identified two BamA-like proteins, LIC11623 and LIC12254, which are conserved across pathogenic *Leptospira* species and are predicted to contain surface-exposed MHC-II binding epitopes. While more work is needed here to validate these targets, they represent promising candidates for inclusion in vaccine development against *Leptospirosis*.

Finally, a reverse-vaccinology approach found potential vaccine candidate proteins in the genome of the fish pathogen *Vibrio anguillarum* [105]. This study identified BamA as a potential vaccine target in *Vibrio anguillarum*, citing BamA conservation across *Vibrio* species. Since BamA was identified as a potential vaccine target in *V. anguillarum* and is conserved across *Vibrio* species, this finding can be extended to human pathogens within the *Vibrio* species.

## 7. Summary and Future Outlook

**Figure 3:**
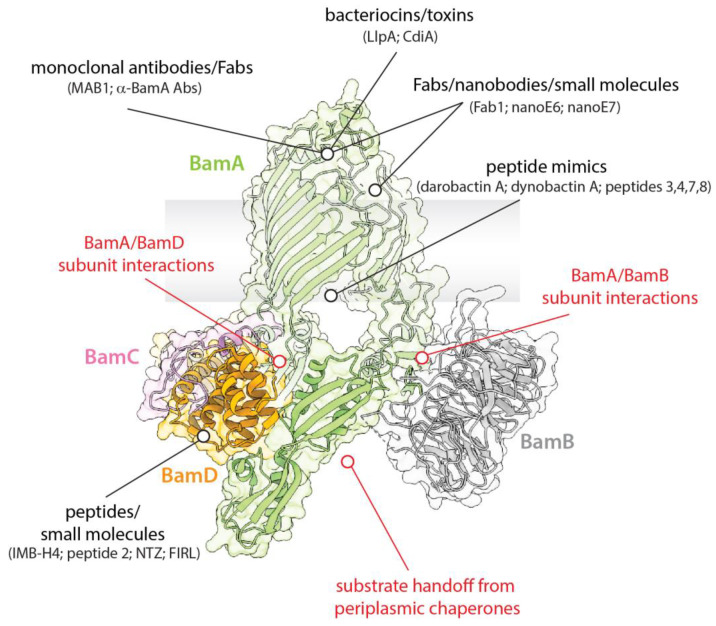
BAM as a promising therapeutic target. The essential role of BAM makes it a promising target for both the development of vaccines and for the discovery of new antibiotics. Regions of BAM that have been successfully targeted are indicated in black, with other interactions that additionally be targeted are indicated in red. Given that BAM is known to be highly dynamic, intermediate states during function would present additional interactions that may also be targeted to disrupt function.

BAM is an essential machinery in Gram-negative bacteria, where it is responsible for the biogenesis of β-OMPs and maintenance of the OM. Given its importance and location on the bacteria surface, it has been an exciting and promising therapeutic target since its initial discovery. Here, we present a summary of recent studies that describe efforts towards understanding the structure and function of BAM and targeting it for therapeutics (Figure 3). The use of small molecules, peptides, and peptidomimetics that target individual components of BAM, such as BamA or BamD, have already proven to be effective; particularly compounds that can directly access BamA from outside the cell. While BamA has been most widely associated with being surface-exposed, studies have convincingly shown that other components can also be found on the outside of the cell. While the mechanism or function for this remains unknown, these observations serve as the basis for the hypothesis that BAM function could also be abrogated by targeting these other components without having to enter the cell. Larger biologics such as antibodies, Fabs, and nanobodies have also been shown to be effective antimicrobials by neutralizing the function of BAM. Additionally, BAM has been implicated in playing a critical role in competition within bacterial communities and the human microbiota by mediating the import of bacteriocins and other toxins across the OM. Whether or not these pathways can be hijacked for therapeutics remains to be determined, but it is a promising prospect given the co-evolution of these systems within bacteria. Lastly, several studies have already targeted BAM for the development of new vaccines, potentially the most useful therapeutic for long-lasting protection. Given the relatively low mutagenesis rate observed in BamA within a bacterial species and between different species of bacteria, identifying suitable epitopes that can elicit a strong immune response with bactericidal activity has the potential to provide generational protection from a host of bacterial pathogens that currently threaten human health in much of the world’s population.

While already an exciting therapeutic target, the discovery of BAM is still relatively new with studies to fully understand its role in β-OMP biogenesis still ongoing. Importantly, the majority of studies on BAM have been performed in *E. coli*, however, more studies are needed to understand the structure and function of BAM across the spectrum of diverse bacteria, particularly in pathogens that pose the highest threat risk to human health. Additionally, better in vitro assays are needed to monitor BAM function to assist in the discovery of additional compounds and scaffolds from high-throughput screening studies that can be further optimized and engineered into promising antibiotics. Similarly, more studies are needed to determine the vaccine potential of BAM and its individual components, which has thus far been limited to only a few studies in select bacteria. Broader studies on a diverse pool of bacterial targets would provide a more informative assessment to determine if BAM indeed has promise as a universal target for future vaccine development.

## Figures and Tables

**Table 1:** Important mutations that disrupt BAM function. Summary of select mutants in Bam proteins that disrupted function and/or led to enhanced sensitivity to existing antibiotics (BamA (formerly called YaeT); BamB (formerly called YfgL); BamC (formerly called NlpB); BamD (formerly called YfiO); BamE (formerly called SmpA)).

	Bam Mutant	Mutation	Effect	Publication
1	BamA	BamAΔR64	Slight sensitivity to vancomycin and rifampin	Bennion et al., 2010. [36]
2	BamA	46 N-terminal insertions into POTRA domains and linker, 41 insertions into β-barrel	Variable vancomycin sensitivity	Browning et al., 2013. [37]
3	BamA	BamA G667V, T671A, R666C	Resistance to LlpA	Ghequire et al., 2018. [38]
4	BamA	BamA E470K	Resistance to MRL-494	Hart et al., 2019. [39]
5	BamA	BamA G429R, G429V, T434A, Q445P, A705T, G433D/E435K/F394V	Resistance to darobactin	Imai et al., 2019. [40]
6	BamA	G429V, G807V, E435K, Q445P, Q445P/T434A	Increased sensitivity to darobactin	Kaur et al., 2021 [41]
7	BamA	BamA F494L	With LptD Y721D, decreased vancomycin sensitivity; in WT, increased vancomycin sensitivity in nutrient-depleting conditions	Lee et al., 2018. [42]
8	BamA	R641E, Δ641RGF643, R641A/G642A/F643A, R641A/G642A, R641A/F643A, G642A/F643A	Increased sensitivity to vancomycin and rifampin	Leonard-Rivera et al., 2012. [43]
9	BamA	G807A, G807V, G807F	G807A, G807V, G807F: increased sensitivity to rifampicin; G807V, G807F: increased sensitivity to vancomycin	Lundquist et al., 2018. [44]
10	BamA	BamA D703Y	Decreased sensitivity to colistin	Luther et al., 2019. [45]
11	BamA	E554Q, H555Y, E554Q/H555Y, L6 deletion	E554Q, H555Y, E554Q/H555Y: decreased sensitivity to MAB1; L6 deletion: decreased sensitivity to MAB2	Storek et al., 2018. [46]
12	BamA	BamA G771A, F738, V660A/R661A, V660A/R661A-LL (loop-to-lumen disulfide bond)	A strain lacking BamA: G771A: hypersensitivity to rifampicin; Strain lacking DegP-BamA G771A, F738: resistant to rifampicin, V660A/R661A: sensitive to rifampicin; (BamA V660A/R661A)-LL: decreased sensitivity to rifampicin	Wzorek et al., 2017. [47]
13	BamA	BamA G429, G809, L501Q, P782, G429V/G807V	Resistance to dynobactin	Miller et al., 2022. [48]
14	BamB	ΔBamB, BamB D227A, D229A, L173S/L175S/R176A	ΔBamB: increased sensitivity to amoxicillin; ΔBamB, D277A, and L173S/L175S/R176A: increased sensitivity to vancomycin, erythromycin, and bacitracin; ΔBamB, D277A, D229A, and L173S/L175S/R176A: increased sensitivity to rifampin, flumequine, and enrofloxacin	Namdari et al., 2012. [49]
15	BamB	recessive LOF mutations in *yfgML* locus via independent element insertions	*yfgML:* resistance to bile salts, chlorobiphenyl vancomycin (CBPV)	Ruiz et al., 2005. [50]
16	BamB	ΔBamB	Increased sensitivity to VUF15259	Steenhuis et al., 2019. [51]
17	BamB	S172-A180 amino acid substitutions (scramble 1 & 2), L173S, L175S, R176A, L173S/L175S, L173S/R176A, L175S/R176A, L173S/L175S/R176A, YfgL(D227A)-His_6_	Scramble 1 and 2: vancomycin hypersensitivity; R176A and either L173S or L175S: vancomycin sensitivity; L173S & L175S & R176A: vancomycin hypersensitivity; YfgL(D227A)-His6: slight increase in vancomycin sensitivity	Vuong et al., 2008. [52]
18	BamE	*omlA:* 170 bp insertion mutation via single recombination	Increased sensitivity to novobiocin, coumermycinA1, chloramphenicol, SDS, and menadione	Fuangthong et al., 2008. [53]
19	BamE	C20G, I32G, Q34G/C, G35C, N36G/C, Y37G, L38G, I46G, V55G, L59G, M64G/C, D66G, F68G/C, W73G, F74G, Y75G/C, V76G, R78G, Q88C, L91G, L93G, F95G/C, L101G	Increased sensitivity to vancomycin	Knowles et al., 2011. [54]
20	BamE	mutant strains: 6B- producing lesser amounts of OmlA (BamE) protein, 3A- lacking a functional *omlA* gene	6B & 3A: increased sensitivity to SDS, deoxycholate 3A: increased sensitivity to nalidixic acid, rifampin, novobiocin, and chloramphenicol	Ochsner et al., 1999. [55]
21	BamE	*smpA* (strain lacking BamE)	Increased sensitivity (4-fold) to rifampin and cholate (2-fold); lethality on media with 0.5% SDS and 1 mM EDTA	Sklar et al., 2007. [56]
22	BamE	BamE deletion	Increased sensitivity to vancomycin	Volokhina et al., 2009. [57]
23	BamF	ΔBamF	Increased sensitivity to TritonX-100, SDS, nalidixic acid, rifampicin, vancomycin, and erythromycin	Anwari et al., 2012. [58]
24	BamA, BamB, BamC, BamE	ΔBamB, ΔBamC, ΔBamE, *bamA101*, BamA H555Y, V322A, P518L, T571M, G575D, G575S	ΔBamB, ΔBamC, ΔBamE, *bamA101*: increased sensitivity to MAB1; BamA H555Y, V322A, P518L, T571M, G575D, G575S: resistance to MAB1	Storek et al., 2019. [59]
25	BamA, BamB, BamD, BamE	mutant strain *bamA101*, ΔBamB, ΔBamC, BamD L13P	Mutant strain bamA101, BamD L13P: significantly increased sensitivity to batimastat; ΔBamB, ΔBamC: slightly increased sensitivity to batimastat	Konovalova et al., 2018. [60]
26	BamA, BamD	*bamA101* (mutant strain with lower BamA expression)*,* BamD_RBS_, BamD_SS_	Sensitivity to bile salts and SDS that is increased at temperatures lower than 37 °C	Mahoney et al., 2016. [61]
27	BamB, BamC	*ΔyfgL* (BamB deletion) *ΔnlpB* (BamC deletion)	*ΔyfgL* eliminated by kanamycin, increased sensitivity to SDS and novobiocin; *ΔnlpB* increased sensitivity to kanamycin	Onufryk et al., 2005. [62]
28	BamB, BamC, BamE	bamB::kan, ΔBamC/ΔBamE	bamB::kan, ΔBamC/ΔBamE: increased sensitivity to bacitracin, erythromycin, novobiocin, rifampin, and vancomycin	Rigel et al., 2012. [63]
29	BamC, BamD	BamC: insertion at codon 41 (*nlpB::kan*); BamD: insertion at codon 227 (*yfiO::kan*)	yfiO::kan allele caused lethality on a BamB LOF allele yfgl8 background; nlpB::dan yflG8 double mutants had irregular colony morphology when exposed to kanamycin	Wu et al., 2005. [15]

**Table 2:** Summary of small molecules and biologics targeting BAM. List of select small molecules, peptides or peptide mimics, antibodies, Fabs, and nanobodies that disrupt BAM function by targeting specific Bam proteins.

Class of Antimicrobial	Name	Source	Cellular Target	MIC	Ref.
Small Molecule	VUF 15259		Autotransporter (AT) pathway	N/A	Steenhuis et al., 2019. [51]
	Nitazoxanide (NTZ)		BamB, BamE, BamD	N/A	Psonis et al., 2019. [68]
	MRL-494		BamA (Gram-negatives); Cytoplasmic membrane integrity (Gram-positives)	25 μM (*E. coli* JCM320)	Hart et al., 2019. [39]
	IMB-H4		BamA, BamD	4 μg/mL (E. coli ATCC 25922)	Li et al., 2020. [69]
Peptide/Protein					
Antibodies	MAB1 (monoclonal antibody)	Mouse/rat	BamA	N/A	Storek et al., 2018. [46]
	anti-BamA monoclonal antibodies	Rat	BamA	N/A	Vij et al., 2018. [70]
Fabs/Nanobodies	Fab1		BamA	N/A	White et al., 2021. [71]
	nanoE6		BamA	N/A	Kaur et al., 2019. [72]
	nanoE7		BamA	N/A	Kaur et al., 2019. [72]
Peptides	JB-95 (β-hairpin peptidomimetic)		possibly BamA or LptD; active against Gram-positives	0.15 μg/mL *E. coli* (E. coli ATCC 25922)	Urfer et al., 2016. [73]
	FIRL (BamD mimic)		BamD	No solo antimicrobial activity; synergizes with existing drugs to lower MIC	Mori et al., 2012. [74]
	Chimeric peptidomimetic antibiotics (peptides 3, 4, 7, 8)		BamA, LPS		Luther et al., 2019. [45]
	Peptide 2 (BamA mimic)	*E. coli*	BamD	N/A	Hagan et al., 2015. [75]
	Antibacterial peptide	*Axinella donnani*	BamA	N/A	Vimala et al., 2015. [76]
	Darobactin A	*Photorhabdus khanii*	BamA	4 μg/mL (*E. coli* MG1655) 2 μg/mL (*E. coli* ATCC 25922)	Imai et al., 2019. [40]
	Dynobactin A	*Photorhabdus australis*	BamA	16 μg/mL (*E. coli* MG1655) 8 μg/mL (*E. coli* ATCC 25922)	Miller et al., 2022. [48]
Lectin-like bacteriocins	LlpA		BamA	N/A	Ghequire et al., 2018; Ghequire et al., 2019. [38,77]

## Data Availability

Not applicable.
